# Complement and MHC patterns can provide the diagnostic framework for inflammatory neuromuscular diseases

**DOI:** 10.1007/s00401-023-02669-8

**Published:** 2024-01-12

**Authors:** Christopher Nelke, Simone Schmid, Felix Kleefeld, Christina B. Schroeter, Hans-Hilmar Goebel, Sarah Hoffmann, Corinna Preuße, Heike Kölbel, Sven G. Meuth, Tobias Ruck, Werner Stenzel

**Affiliations:** 1 Department of Neurology, Medical Faculty, Heinrich Heine University Duesseldorf, Moorenstr. 5, 40225 Duesseldorf, Germany; 2 Department of Neuropathology, Charité-Universitätsmedizin Berlin, Corporate Member of Freie Universität Berlin, Humboldt-Universität zu Berlin, Berlin Institute of Health (BIH), Charitéplatz 1, 10117 Berlin, Germany; 3 Department of Neurology, Charité-Universitätsmedizin Berlin, Corporate Member of Freie Universität Berlin, Humboldt-Universität zu Berlin, Berlin Institute of Health (BIH), Charitéplatz 1, 10117 Berlin, Germany; 4 Department of Neuropediatrics, Charité-Universitätsmedizin Berlin, Corporate Member of Freie Universität Berlin, Humboldt-Universität zu Berlin, Berlin Institute of Health (BIH), Augustenburger Platz 1, 13353 Berlin, Germany; 5 Department of Neuropaediatrics, Klinik für Kinderheilkunde I, Universitätsklinikum Essen, Essen, Germany; 6 Leibniz Science Campus Chronic Inflammation, Berlin, Germany

**Keywords:** Complement, Major histocompatibility complex, Myasthenia gravis, Myositis, Muscle dystrophies

## Abstract

Histopathological analysis stands as the gold standard for the identification and differentiation of inflammatory neuromuscular diseases. These disorders continue to constitute a diagnostic challenge due to their clinical heterogeneity, rarity and overlapping features. To establish standardized protocols for the diagnosis of inflammatory neuromuscular diseases, the development of cost-effective and widely applicable tools is crucial, especially in settings constrained by limited resources. The focus of this review is to emphasize the diagnostic value of major histocompatibility complex (MHC) and complement patterns in the immunohistochemical analysis of these diseases. We explore the immunological background of MHC and complement signatures that characterize inflammatory features, with a specific focus on idiopathic inflammatory myopathies. With this approach, we aim to provide a diagnostic algorithm that may improve and simplify the diagnostic workup based on a limited panel of stainings. Our approach acknowledges the current limitations in the field of inflammatory neuromuscular diseases, particularly the scarcity of large-scale, prospective studies that validate the diagnostic potential of these markers. Further efforts are needed to establish a consensus on the diagnostic protocol to effectively distinguish these diseases.

**Supplementary Information:**

The online version contains supplementary material available at 10.1007/s00401-023-02669-8.

## Introduction

Histopathological analysis of skeletal muscle remains the diagnostic benchmark for detecting and distinguishing neuromuscular disorders, even though genetic and biochemical techniques have a well-established place in the diagnostic work-up. Historically, visual analysis of disease pathophysiology evolved from early studies employing light microscopy in the nineteenth century to examination on the subcellular level by contemporary methods such as electron microscopy [99]. Histological analysis was augmented by staining of paraffin-embedded tissue prior to microscopy. Early staining strategies involved visualization by hematoxylin and eosin (HE) or modified Gömöri trichrome, while the emergence of immunohistochemistry (IHC) in the 1980s, as pioneered by the late Kiichi Arahata, Andrew Engel and many others [8], allowed for the targeted analysis of specific proteins and cellular interplays. Together with clinical and laboratory parameters including antibody testing, histopathological analysis provides the framework for the contemporary classification of idiopathic inflammatory myopathies (IIM) [43]. Currently, we recognize dermatomyositis (DM), immune-mediated necrotizing myopathy (IMNM), antisynthetase syndrome-associated myositis (ASyS), and inclusion body myositis (IBM) as prototypical entities across the IIM spectrum [42]. However, this classification is challenged by a substantial clinical and histopathological overlap between different disorders as well as divergent disease outcomes even in individual subgroups of IIM. We and others have previously suggested that combining clinical, serological and morphological parameters in a clinico-seropathological model could enhance the classification of IIMs [10, 11, 43, 87]. Still, exact classification of disease is crucial for determining the individual treatment success, long-term outcomes, and clinical manifestations. Standardization of analytical workflows for muscle pathologies remains challenging as highlighted by a recent survey across 61 neuromuscular centers reporting highly variable diagnostic algorithms [91]. To improve standardization, the EURO-neuromuscular diseases (NMD) pathology working group suggested a harmonized workflow aiming to standardize the classification of muscle diseases, including IIMs [91]. While comprehensive, substantial resources and expertise are required for diagnostic algorithms directed at muscle pathologies often only available in highly specialized neuromuscular centers. However, a considerable number of affected patients are likely to live in low- and middle-income countries [28, 48]. The limitations imposed by these health care systems make it difficult to provide comprehensive, in-depth histopathological analysis as routine diagnostic approaches.

To address this unmet need and to improve the diagnostic acuity of histopathology in a resource-limited setting, we here propose to distinguish IIM according to the current myopathological classification based on interpretation of major histocompatibility complexes classes I and II (MHC cl. I and -II) and complement patterns. This approach centers on visualizing MHC and complement, reflecting current knowledge of immunological concepts of disease. We also extend this framework to the analysis of skeletal muscle tissues of certain hereditary myopathies and the neuromuscular synapse, highlighting recent advances in those fields. In the following section, we will discuss the immunological background of MHC and complement in neuromuscular autoimmunity.

## Immunological background

The MHC system is responsible for presentation of antigens. MHC class (cl.) I is present on all nucleated cells and presents immunogenic peptides salvaged from intracellular proteasomes to immune cells. Concurrently, MHC cl. II is employed by antigen-presenting cells (APC) to interact with CD4 T cells, eventually orchestrating the development of specific effector cells. Variations and functions including complexity of antigen presentation by MHC molecules have been extensively studied given their association with autoimmune, infectious and metabolic diseases among others [39, 54].

In the context of muscle pathology, MHC positivity emerged as a histopathological feature shared across the IIM spectrum. In our experience and that of others, the majority of muscle samples derived from IIM patients stain positive for MHC cl. I [62]. Although sarcolemmal MHC cl. I expression is highly characteristic for IIM, MHC cl. I can be observed in other muscle pathologies such as muscular dystrophy (~ 11% of cases) and others [62]. The close association of MHC cl. I expression in IIM has fostered the view that MHC cl. I participates in the pathogenesis of the latter. The degree of MHC cl. I expression may coincide with the degree of muscle inflammation, but may also occur at distance from cellular infiltrates [68, 87]. Importantly, while MHC cl. I is usually not detected in healthy muscle tissue, regenerating myofibers may demonstrate sarcoplasmic MHC cl. I positivity. Further, the pattern and distribution of MHC cl. I varies considerably within different regions of fascicles and between different entities. Mechanistically, stimulation of human muscle cells by pro-inflammatory cytokines induces MHC cl. I and MHC cl. II expression allowing for antigen presentation to T cells [31]. In a recent mouse model, muscle-specific upregulation of MHC cl. I resulted in development of myositis—partly reminiscent of disease patterns in humans [52]. Intriguingly, autoantibodies directed against the histidyl-tRNA synthetase were detected in this model suggesting that the apparently non-specific upregulation of MHC cl. I may induce a highly specific autoimmune event as consequence. While convincing data on the association between MHC and muscle inflammation exists, our mechanistic understanding of how MHC complexes might instigate and sustain IIM is limited. MHC cl. I and MHC cl. II overlap in many characteristics such as a high level of polymorphism, a similar structural composition, a genetic location in one locus and similar functions as antigen-presenting molecules [54]. Notably, MHC cl. II alleles emerged as convincing genetic markers for the development and severity of autoimmune diseases, including IIMs [49, 74]. MHC cl. II molecules are primarily expressed by APCs such as dendritic cells, macrophages and B cells. However, MHC cl. II and its upstream regulator CIITA can be induced by interferon-γ promoting the development of immune-mediated diseases [54].

Recently, sarcolemmal and capillary complement deposits were described as histopathological features of IIM with complement as a varying feature across the spectrum of IIM with ASyS, DM and IMNM as notable examples [2]. Traditionally, complement has been regarded as a serum-effective response against microbes supporting the innate and adaptive immune responses. Over the past decade, this view has changed profoundly. It became clear that the complement system regulates immunological and metabolic functions beyond the elimination of microbial threats [56, 69]. While historically regarded as a linear system cumulating in the formation of the membrane attack complex (MAC) and target cell lysis, the contemporary view assumes that branches of the complement system act concurrently, thereby fine-tuning the immune response in relation to the offending threat [69]. Extending the current knowledge of complement to IIM, its activation is thought to respond to myofiber destruction with necrotic muscle fibers often displaying deposits of terminal complement compounds [19]. Exemplified in certain subtypes of DM, capillary endothelium is targeted by the MAC resulting in the release of proinflammatory cytokines [40]. Inflammation is amplified by a cellular response further promoting a proinflammatory micromilieu. The release of cytokines also links complement activation to the upregulation of MHC cl. I as myofibers respond to soluble factors such as tumor necrosis factor α (TNFα) by expression of MHC cl. I [19, 75]. While the association of complement and muscle inflammation is convincing, it is challenging to pinpoint the pathophysiological role of complement in mediating IIMs. One theory, although controversial, suggests that autoantibodies may trigger the complement cascade via the classical complement pathway resulting in complement-mediated myofiber injury [2]. However, this line of argumentation is challenged by a number of arguments: (1) Complement deposition is also observed in hereditary muscle disease such as muscular dystrophy (e.g., LGMDR2) [81], thereby acting independently of any known autoantibodies. (2) Antibodies directed against structures such as the signal recognition particle (SRP) or the HMG-CoA reductase (HMGCR), as seen in IMNM, are non-specific as these antigens are ubiquitous cytoplasmic proteins expressed in several tissues. Selective muscle fiber damage would require a specific immune mechanism that directs complement specifically to muscle or capillaries in muscles, but not to all the other tissues [64]. (3) Complement activation is also observed, albeit more rarely, in seronegative IIM such as IBM patients without detectable antibodies [55], or seronegative IMNM [55]. Conversely, it is conceivable that the respective autoantibodies have not yet been identified. Although complement plays a prominent role in immune mechanisms in IMNM [3], complement inhibition did not prove effective in a recent phase 2 trial [46].

Taken together, the pathophysiological role of MHC and complement including their specific interactions in IIM requires further research efforts. Interestingly, the patterns formed by MHC and complement are consistently distinct across different IIM entities suggesting that pathophysiological characteristics might be reflected in these morphological differences.

In this review, we will focus on the diagnostic value of MHC and complement patterns and how their signatures may be employed to distinguish inflammatory muscle diseases from each other and from potential differential diagnoses.

## MHC and complement patterns in idiopathic inflammatory myopathies

The various subtypes of IIM are characterized by distinct pathomorphological presentations, which were extensively described in the past decades [10, 87]. Here, we will first aim to provide an overview of MHC cl. I, -II and complement patterns across IIM subtypes (Table 1). This simplified algorithm might improve diagnostic strategies in non-specialized centers or in resource-limited settings.

**Table 1:** Overview of MHC class I, MHC class II, and complement in inflammatory muscle diseases

Disease	MHC class I	MHC class II	Complement (C5b-9)	Ref
Idiopathic inflammatory myopathies				
Immune-mediated necrotizing myopathy (SRP and HMGCR)	+ /++	−	+ (sarc)	[2, 3, 67, 98, 100]
TIF1γ dermatomyositis	+++ (pf)	−	++ (cap)	[45, 84]
Mi2 dermatomyositis	++ (pf)	+	+ (sarc)	[45, 59, 84]
NXP2 dermatomyositis	+++ (pf)	+/−	++ (cap)	[45, 84]
MDA5 dermatomyositis	+	−	+ (cap)	[45, 84]
SAE dermatomyositis	+	+/−	+/− (cap)	[45, 84, 85]
Anti-synthetase syndrome	++ (pf)	++ (pf)	+ (sarc)	[6, 60, 68, 83]
Inclusion body myositis	+++	+++	−	[20, 21, 33, 62, 73]
Overlap myositis*	+	+	−	[10, 41, 70]
Other immune-mediated muscle diseases				
Eosinophilic fasciitis	+	+	−	[63, 89]
Immune checkpoint inhibitor-related myositis	+	++	−	[1, 76]
Scleromyositis and MMCP	++	+/−	+/− (sarc)	[30, 80]
Hereditary myopathies				
LGMDR1	−	+	+/−	[9]
LGMDR2	+	−	++ +	[15, 37]
LGMDR9	+	+	−	[15, 37]
LGMDR12	+	−	+/−	[14]
Duchenne muscular dystrophy	+	+	+/−	[7, 47]
Autophagic vacuolar myopathies	++ (sarc)	?	++ (sarc)	[18, 23, 44, 53]

Overview of MHC class I, MHC class II and complement in inflammatory muscle diseases. The intensities of the corresponding marker are indicated by + and – with +++ indicating very strong expression,  ++  strong expression, + mild/infrequent expression, − absence of the corresponding marker and  +/− indicating varying degrees of expression. ? indicates a marker with inconclusive data regarding its diagnostic value

*cap* on the capillary; *LGMD* limb girdle muscular dystrophies; *MDA5* melanoma differentiation-associated protein 5; *MMCP* minimal myositis with capillary pathology; *NXP2* nuclear matrix protein 2; *pf* perifascicular; *SAE* small ubiquitin-like modifier-1 activating enzyme; *sarc* sarcolemmal; *TIF1γ* transcriptional intermediary factor 1 gamma

*The histopathological features of overlap myositis are highly heterogeneous and dependent on the clinical context and autoantibody status

### Immune-mediated necrotizing myopathy

**Fig. 1:**
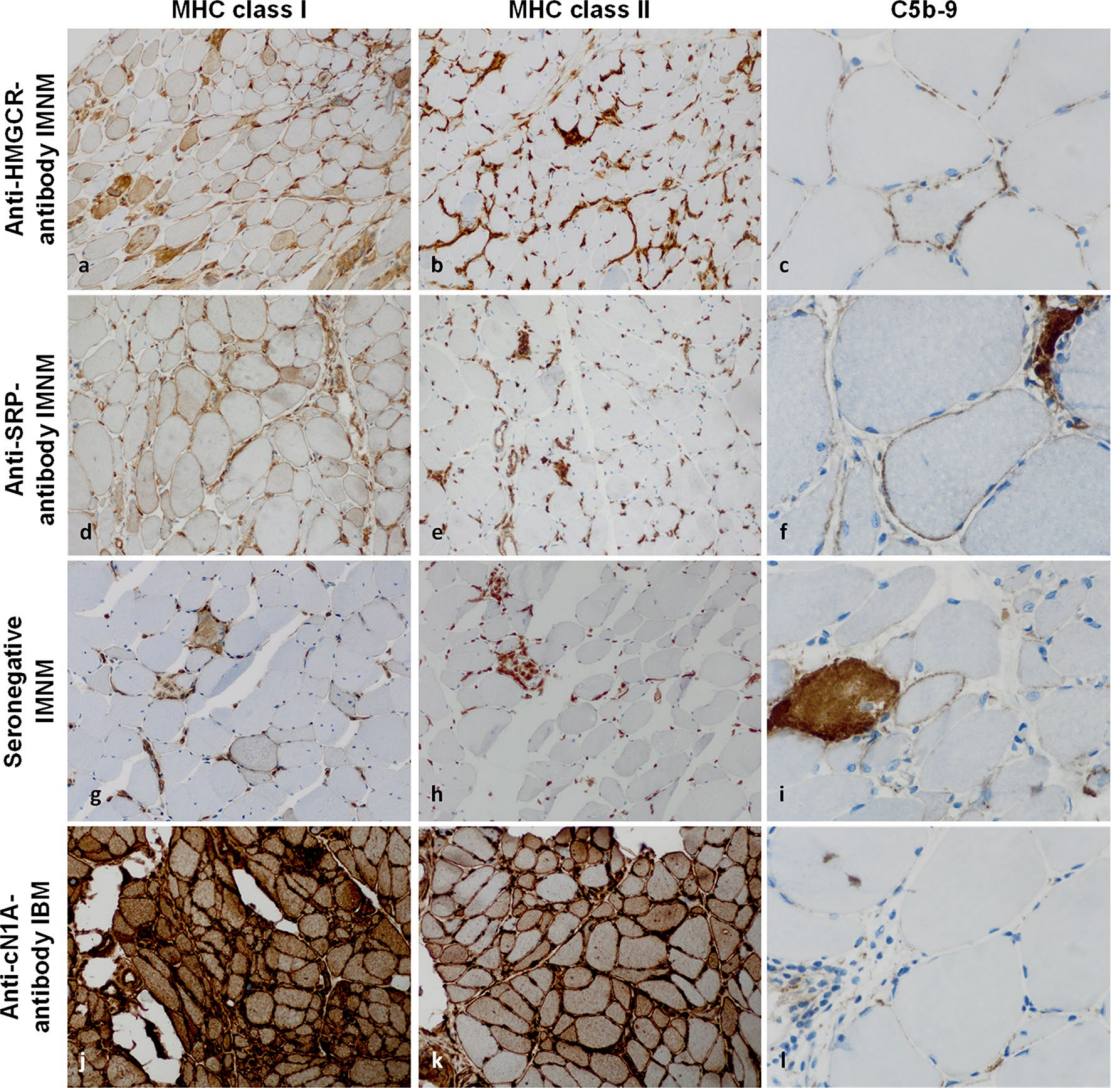
MHC class I, -II, and complement patterns in immune-mediated necrotizing myopathy and inclusion body myositis. In immune-mediated necrotizing myopathy (IMNM), the sarcolemma of myofibers is frequently positive for MHC class I in a diffuse distribution pattern (**a, d, g**). MHC class II is consistently negative on the myofiber sarcolemma (**b, e, h**). Macrophages consistently demonstrate diffuse MHC class II positivity in the endomysium. Capillaries are physiologically positive for MHC class I and MHC class II. In response to inflammation, MHC levels are typically upregulated. This phenomenon is non-specific across IIMs and applies to all figures shown in this manuscript. Terminal complement (C5b-9) is often positive on the sarcolemma of non-necrotic fibers in IMNM with a fine punctuated pattern (**c, f, i**). In inclusion body myositis (IBM), MHC class I and II are positive in a strong and diffuse pattern on the sarcolemma (and the sarcoplasm) without perifascicular staining pattern (**j, k**). C5b-9 may be detected non-specifically on fibroblasts, but not on the capillaries or the sarcolemma of myofibers in the majority of IBM cases (**l**). (**c**, **f**, **i**, **l**) are 600× magnification to exemplify the punctuated pattern in IMNM; all other images are 200 × magnification. *IIM* idiopathic inflammatory myopathy; *MHC* major histocompatibility complex

Recently, we characterized complement activation in anti-signal recognition particle (SRP)- and anti-HMG-CoA reductase (HMGCR)-antibody positive IMNM [2]. Focusing on complement patterns, it is noteworthy that biopsies from IMNM patients display a diffuse distribution of myofiber necrosis and regeneration with numerous fibers showing features of myophagocytosis [2]. The sarcolemma stains positive for scattered MHC cl. I, while MHC cl. II is usually not detected on the sarcolemma. Here, complement deposits may be detected on the sarcolemma of non-necrotic myofibers with a punctuated pattern, likely derived from the classical complement pathway (Fig. 1a–i). Additionally complement is detectable sarcoplasmically in association with muscle fiber necrosis, but this is non-specific and may occur in myofiber necrosis irrespective of the underlying etiology. Conversely, a suspected case of IMNM that demonstrates MHC cl. II positivity on myofibers might hint toward an overlap myositis (see below). Interestingly, we observed a positive correlation between sarcolemmal complement deposits and muscle fiber necrosis in IMNM [2]. This association might implicate complement in mediating muscle fiber destruction. Of note, the current morphological IMNM classification requires the regular presence of sarcolemmal complement deposits, while myofiber necrosis is excessively variable and hence unlikely to constitute a dominant histological feature [2]. Based on our finding of considerable variation of muscle fiber necrosis, regeneration and inflammation, we suggest including the entire histopathological features of IMNM to capture the full range of disease presentation, particularly in those patients without detectable autoantibodies [3, 5, 64]. These criteria were recently employed for a phase 2 study of IMNM investigating the C5 inhibitor zilucoplan [46].

### Inclusion body myositis

Sarcolemmal MHC cl. I, and -cl. II are consistently detected in IBM, and conversely the absence of MHC staining on sarcolemma is a strong argument against the diagnosis of IBM. MHC patterns are distinct from the above-mentioned patterns in IBM as they are not restricted to the perifascicular regions. Instead, both MHC cl. I and MHC cl. II are found in close proximity to areas of endomysial inflammation with intensities increased where lymphomonocytic cell infiltrates are particularly prominent (Fig. 1j, k) [33, 55]. In contrast, complement deposition is non-specific and may be restricted to areas of endomysial fibrosis, while sarcolemma or capillaries do not consistently stain positive for complement (Fig. 1l) [55].

### Dermatomyositis

**Fig. 2:**
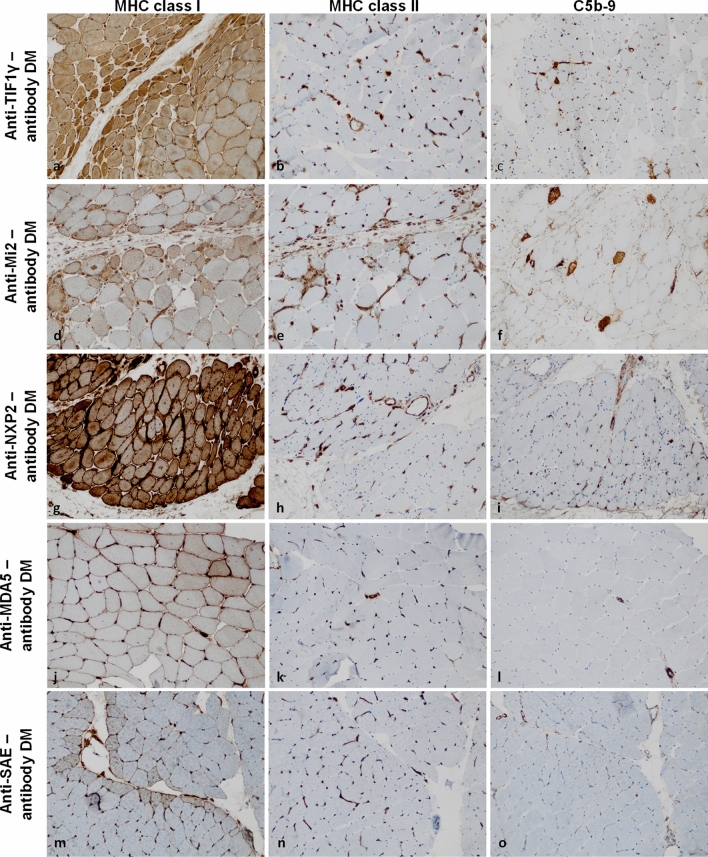
MHC class I, -II, and complement patterns in dermatomyositis subtypes. In dermatomyositis (DM), the sarcolemma of myofibers is positive for MHC class I in a strong perifascicular to a weak centrofascicular distribution (**a, d, g, j, m**). MHC class II is consistently negative on the sarcolemma of myofibers (**b, h, k, n**) with the exception of anti-Mi2-antibody DM (**e**). Here, myofibers in the perifascicular region are infrequently and relatively weakly positive for MHC class II. Terminal complement (C5b-9) is positive on the capillaries in perifascicular regions in anti-TIF1γ-antibody and anti-NXP2-antibody DM (**c, i**). C5b-9 is also found on the sarcolemma of non-necrotic myofibers in perifascicular regions in anti-Mi2-antibody DM (**f**). In anti-MDA5-antibody and anti-SAE-antibody DM, C5b-9 may be positive on individual capillaries or the sarcolemma of few myofibers without a specific pattern (**l, o**). (Original magnification × 200). *MDA5* melanoma differentiation-associated protein 5; *MHC* major histocompatibility complex; *NXP2* nuclear matrix protein 2; *SAE* small ubiquitin-like modifier-1 activating enzyme; *TIF1γ* transcriptional intermediary factor 1 gamma

While certain histopathological features are conserved across DM subtypes, the specific presentation of each subtype is highly characteristic for the associated autoantibodies. This concept might be best exemplified by DM mediated by antibodies against transcription intermediary factor 1 gamma (TIF1γ). Here, on a background of perifascicular fiber atrophy, MHC cl. I is strongly detected on the sarcolemma and the sarcoplasm as well, exhibiting a gradient toward the centrofascicular region in many fascicles [8, 23] (Fig. 2a). In contrast, MHC cl. II is negative on the sarcolemma, but positive on the many macrophages in the peri- and the endomysium and also positive physiologically on capillaries (Fig. 2b). Terminal complement is prevailing on capillaries rather than on the sarcolemma of myofibers, especially in severely affected muscle regions associated with capillary loss (Fig. 2c), and prominent vascular endothelial growth factor (VEGF) signaling [66]. Together, these features create a highly specific pattern, characteristic of anti-TIF1γ-antibody mediated DM. These characteristics are associated with the presence of coincident cancer in 40–50% of adult cases [34, 45, 91], whereas juvenile anti-TIF1γ-antibody DM is unlikely to coincide with cancer.

A different pattern is observed in DM mediated by anti-Mi-2-antibodies. Here, the pathological perifascicular pattern consists of atrophic fibers in perifascicular regions, interleaved with myofiber necrosis. The latter may also occur in the centrofascicular regions and is not strictly confined to the perifascicular area. MHC cl. I is strongly positive on the sarcolemma of perifascicular myofibers with a gradient toward the centrofascicular region (Fig. 2d), while MHC cl. II is only weakly detected on the sarcolemma on sparse perifascicular muscle fibers while physiologically positive on capillaries. C5b-9 may be observed on the sarcolemma predominantly on atrophic muscle fibers of the perifascicular area but not significantly on capillaries. As such, the sarcoplasm of necrotic myofibers often stains positive for complement in anti-Mi2-antibody DM (Fig. 2e) [10, 85].

In anti-nuclear matrix protein 2 (NXP2)-antibody positive DM, MHC cl. I is characteristically detected with perifascicular positivity on sarcolemmal membranes and occasionally on entire fascicles, while MHC cl. II is absent from the sarcolemma (Fig. 2g, h). C5b-9 may be detected on capillaries as well as on the sarcolemma of myofibers (Fig. 2i). A rare but unique feature is the presence of regional muscle fiber necrosis affecting parts of an entire fascicle encountered in some cases of anti-NXP2-antibody DM (not shown here) [10, 85].

Anti-melanoma differentiation-associated gene 5 (MDA5)-antibody-mediated DM displays milder affection of skeletal muscle tissues, and the perifascicular pattern is usually only found in some areas of the specimen. MHC cl. I may be detected on the sarcolemma of some but not all fibers (Fig. 2j), while sarcolemmal MHC cl. II is usually not observed (Fig. 2k). Complement deposits either on scarce sarcolemmal membranes or on capillaries are only rarely encountered (Fig. 2l) [4, 10, 85].

Finally, the pattern of anti-small ubiquitin-like modifier activating enzyme (SAE)-antibody DM has not been systematically characterized and is subject of current research [22, 87]. Our own unpublished data point toward a pattern that is similar to the one described in anti-MDA5-antibody DM with mild MHC cl. I (Fig. 2m), absence of MHC cl. II on the sarcolemma of perifascicular myofibers (Fig. 2n), and relatively scarce complement deposits on sarcolemma and on capillaries (Fig. 2o).

Broadly, complement is more frequently observed on capillaries than on the sarcolemma of myofibers in DM. The complement deposits on capillaries correlate with muscle damage characteristic of DM and capillary damage can be substantiated by ultrastructural visualization of tubuloreticular inclusions in vascular endothelium [6, 10].

Finally, while this review is focused on patterns of complement and MHC, it should be highlighted that the interferon 1 response has emerged as a driver of DM pathogenesis, likely contributing to the characteristic muscle damage and capillary involvement seen in this disease [32]. The binding of the type 1 interferons to their target receptors induces the transcription and translation of a gene signature consisting of type 1 interferon-inducible genes, such as myxovirus resistance protein A (MxA) (Supplemental Fig. 1). Following this line of argumentation, recent studies highlighted the diagnostic value of MxA staining by immunohistochemistry reflecting the underlying type 1 interferon response in DM [92, 93]. Indeed, two comparative studies recently demonstrated that the sensitivity and specificity of MxA to distinguish between DM and other forms of IIM range between 71% and 77%, and 98 and 100%, respectively [92, 93]. This renders MxA staining particularly valuable for improving the diagnostic confidence when differentiating between anti-Mi2-antibody mediated DM and cases of ASyS as these entities may closely resemble each other.

### Antisynthetase syndrome myositis

**Fig. 3:**
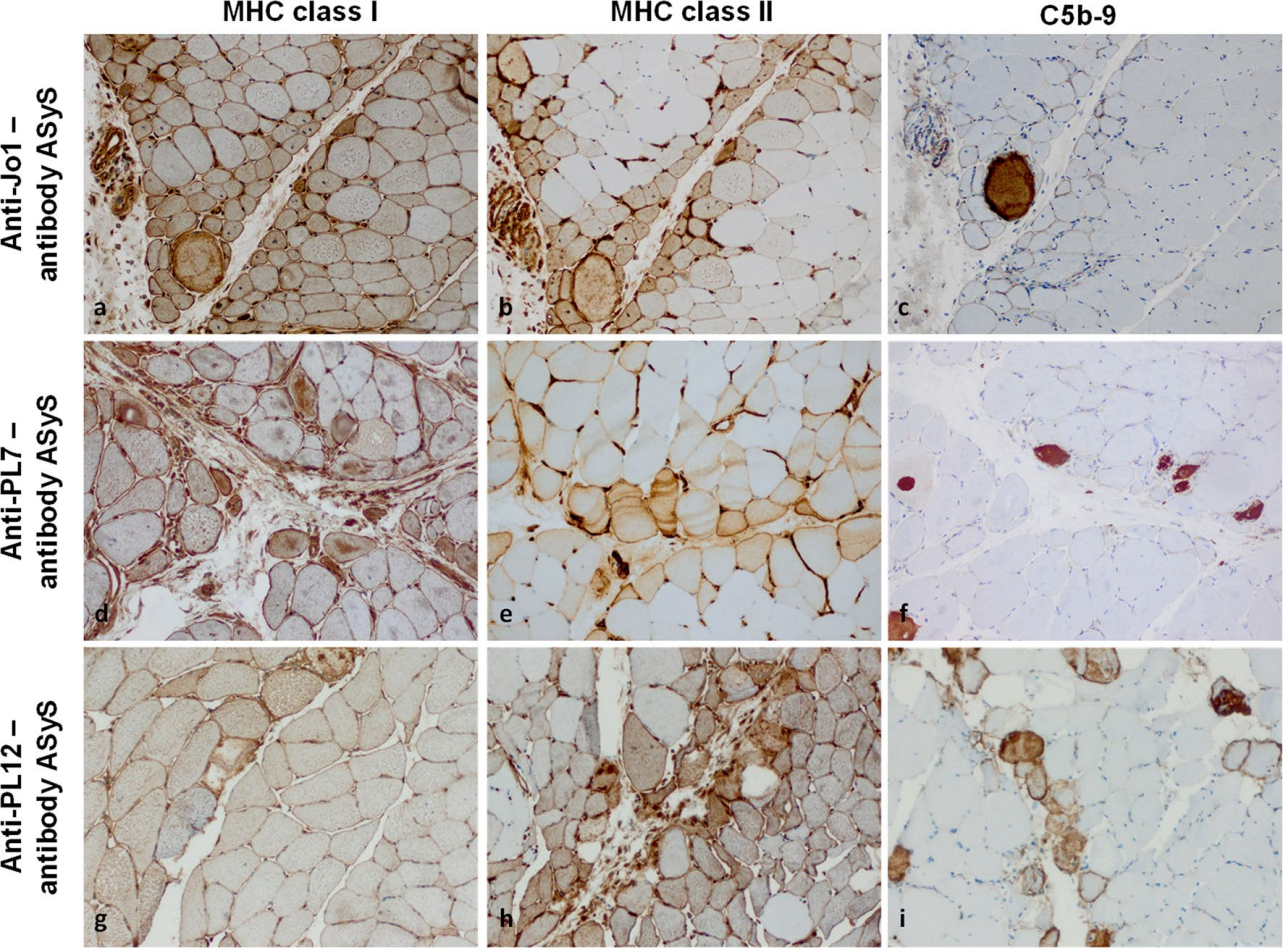
MHC class I, -II, and complement patterns in subtypes of anti-synthetase syndrome. In anti-synthetase syndrome (ASyS), the sarcolemma of myofibers is often positive for MHC class I (**a, d, g**) and class II (**b, e, h**) in a strong perifascicular to weak centrofascicular pattern. Terminal complement (C5b-9) is positive on the sarcolemma of non-necrotic myofibers predominantly in the perifascicular region (**c, f, i**). A variable number of myofibers shown sarcoplasmic staining for C5b-9 highlighting the perifascicular necrotizing pattern of ASyS. (Original magnification × 200). *MHC* major histocompatibility complex

Conversely, the pathomorphology of ASyS is largely consistent across the currently recognized antibody-subtypes (Fig. 3) [68, 83]. Recent research suggested two additional ASyS-associated antibodies (anti-lys, anti-val) directed against transfer-RNA [77, 97]. This observation is in line with recent data from our laboratory reporting only relatively subtle differences of inflammatory molecules on the molecular level across ASyS antibody groups [68]. ASyS is identified by a perifascicular pathology characterized by myofiber necrosis aligned with both atrophic and hypertrophic muscle fibers differing from perifascicular atrophy patterns in DM. Here, MHC cl. I and MHC cl. II are both detected on the sarcolemma of myofibers with a decreasing gradient of intensity toward the centrofascicular regions (Fig. 3a, d, g). Specifically, MHC cl. II might more characteristically highlight this pattern as compared to MHC cl. I (Fig. 3b, e, h). In respect to complement, C5b-9 is frequently detected on the sarcolemma of perifascicular fibers, but only rarely on capillaries, and the pattern of perifascicular necrosis visualized by sarcoplasmic C5b-9 is variably intense from mild (Fig. 3c), intermediate (Fig. 3f) to strong (Fig. 3i) across ASyS samples [82, 83].

### Eosinophilic myofasciitis

**Fig. 4:**
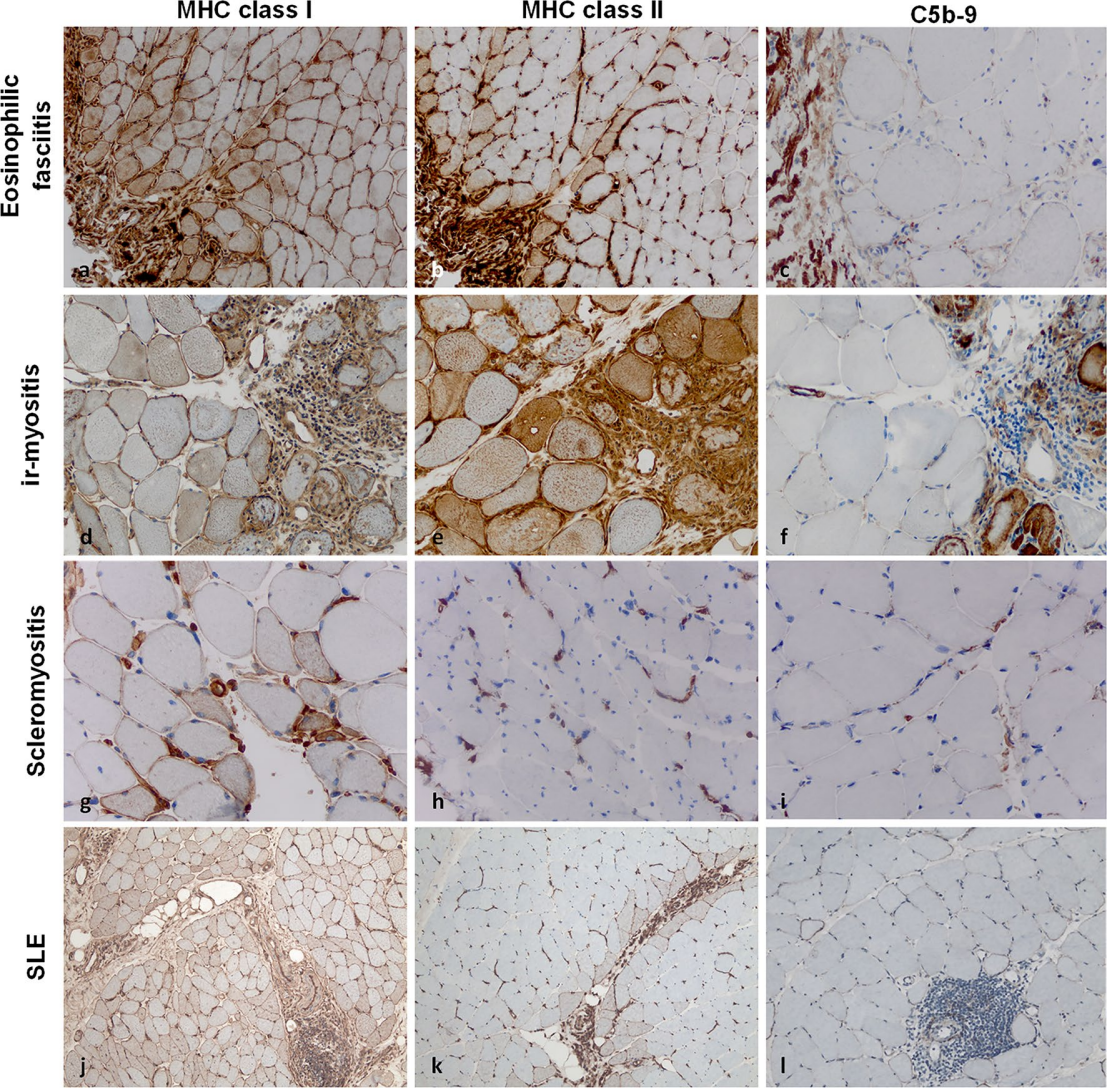
MHC class I, -II, and complement patterns in eosinophilic fasciitis, immune checkpoint inhibitor-related myositis, scleromyositis and systemic lupus erythematosus. In eosinophilic fasciitis, myofibers are positive for MHC class I on the sarcolemma, while MHC class II is observed with a perifascicular (strong) and centrofascicular (weak) pattern (**a, b**). Terminal complement (C5b-9) is negative on the sarcolemma and on capillaries, even in proximity to areas of inflammation (**c**). Immune checkpoint inhibitor-related myositis (ir-myositis) is characterized by strong positivity of MHC class I and II in areas of inflammation, hence with a focal pattern on the sarcolemma (**d, e**). C5b-9 is observed on necrotic myofibers in a non-specific pattern, but not on capillaries (**f**). In scleromyositis, MHC class I is positive on myofibers around inflammation (**g**). MHC class II is often negative and only observed in few cases sarcolemmaly (**h**). C5b-9 is detected on individual thickened capillary walls with a focally distributed pattern (**i**). In systemic lupus erythematosus (SLE), MHC class I positive myofibers are detectable with a characteristic (“chessboard”) pattern across entire fascicles (**j**) and MHC class II is highlighting myofibres adjacent to the perimysium (**k**). C5b-9-positive complement deposits are detected throughout fascicles and do not accumulate in perifascicular areas. Of note, (**l**) dense lymphomonocytic infiltrates may accumulate in the endomysium but invasion of myofibers is not visible. (Original magnification × 200 for all photomicrographs). *MHC* major histocompatibility complex

Eosinophilic myofasciitis (EF), also called Shulman syndrome, is an entity that affects the muscle fascia interface morphologically, and clinically follows a highly characteristic appearance that differentiates this disease from DM and ASyS. EF is not associated with known autoantibodies. MHC cl. I and cl. II are typically positive on the sarcolemma of perifacicular myofibres adjacent to the epimysial fascia (Fig. 4a, b), while the deeper layers of the muscle tissue show much less or even absence of any sarcolemmal staining [63]. Complement is largely absent on the sarcolemma or capillaries of diseased muscle (Fig. 4c). The detection of eosinophils in the fascia may help distinguishing EF from other immune-mediated fasciitis with associated inflammatory myopathy.

### Immune checkpoint inhibitor-related myositis

In immune checkpoint inhibitor-related (ir) myositis, the currently recognized pattern is muscle fiber necrosis with varying degrees of endomysial macrophage infiltrates and myophagocytosis [76]. Here, lymphocytes, particularly CD8 T cells, are abundantly found in the endomysium. MHC cl. I and cl. II are frequently detected with different patterns and distribution reported (Fig. 4d, e). Systematic studies of complement are currently lacking. Still, necrotic myofibers are frequently encountered in ir-myositis and are highlighted by sarcoplasmic C5b-9 (which is useful but not disease-specific) (Fig. 4f) [90]. The recent detection of three distinct subtypes of ir-myositis (ICI-DM with DM-like features; ICI-MYO1 with abundant inflammation and myocarditis; ICI-MYO2 with necrosis but with little inflammatory features and without myocarditis), revealed that ICI-MYO1 presenting with myocarditis and excessive inflammation had the worst prognosis [65]. It should be noted that ir-myositis associated with myocarditis should be treated aggressively given its high mortality [76].

### Overlap myositis

This heterogeneous group of diseases is clinically characterized by overlap of rheumatological diseases with inflammation of skeletal muscles. Myositis-associated antibodies include anti-Ku, anti-PM/Scl, anti-U1-RNP-antibodies among others. Although heterogeneous, in our experience, these entities often display MHC cl. I and often cl. II on the sarcolemma, pronounced around endomysial infiltrates of immune cells, and sometimes even with a perifascicular distribution, but in contrast to the above-mentioned entities (DM and ASyS) complement deposits are only detected on the sarcolemma of single myofibers without any specific pattern. Although recent focus has been on the morphological description of the IIM sensu* strictu*, overlap myositis presenting with myositis-associated antibodies regularly demonstrate MHC cl. I and cl. II positivity on the sarcolemma, while the quantity and distribution of invading immune cell vary substantially and so does the capillary pathology [80].

Recently, two groups independently described the histopathological features of myositis in patients with systemic sclerosis, thereby raising the concepts of scleromyositis and minimal myositis with capillary pathology (MMCP) [25, 26, 80]. Here, MHC cl. I is detected in a focal pattern linked to areas of endomysial inflammation (Fig. 4g), while MHC cl. II is only rarely found on individual myofibers or absent (Fig. 4h). While capillaries are enlarged and often positive for MHC cl. I and cl. II in many areas of the specimen, complement deposition is non-specifically found on myofibers and capillaries (Fig. 4i).

Additionally, myopathy may also develop in patients with systemic lupus erythematosus (SLE) [13, 88]. About 6% of patients may encounter myopathy during the course of their disease. Histopathologically, SLE myopathy may present with areas of myofiber necrosis and regeneration. Perimysial or endomysial inflammation is found in approximately a third of patients [13, 88]. Importantly, SLE myopathy may mimic the clinical and histopathological manifestation of DM and IMNM constituting a diagnostic challenge (Fig. 4j, k, l). The overlap between SLE myopathy or DM might be due to a shared interferon type 1 response as pathophysiological driver [24]. As such, integration of clinical characteristics, including the antibody status and SLE-specific symptoms, with the histopathological profile is important to effectively distinguish between SLE myopathy and DM.

### Hereditary myopathies

To provide a broader picture of MHC and complement in muscle pathologies, we will also describe patterns in certain hereditary myopathies, aiming to cover relevant entities, since this endeavor has not been undertaken systematically in many of them. Limb-girdle muscular dystrophies (LGMD) are hereditary myopathies, which variably display inflammatory features. As such, sarcolemmal complement deposition is a striking finding on numerous myofibers in dysferlin-related limb-girdle muscular dystrophy R2 (LGMDR2) [9, 95]. Intriguingly, the amount of sarcolemmal complement detected in LGMDR2 may even exceed that in certain entities of IIM. Conversely, MHC cl. I and MHC cl. II staining is only mild or absent on occasional myofibers surrounding lymphocytic infiltrates [9]. In biopsies from patients with limb-girdle muscular dystrophy R12 (LGMDR12) due to *ANO5* gene mutations, muscle inflammation may also be observed as evidenced by myophagocytosis and focal but not diffusely distributed muscle fiber necrosis and regeneration accompanied by scarce lymphocytes. Sarcolemmal MHC cl. I positivity is very mild, while sarcolemmal complement is sparsely detected on single myofibers [14]. In contrast, in muscle biopsies obtained from patients with LGMDR9 related to *FKRP* mutations, we described a focal accumulation of sarcolemmal MHC cl. I, and MHC cl. II on specific myofibers associated with myofiber regeneration. Complement deposits were not found in these biopsies [37]. Sparse sarcolemmal deposits of complement and focal MHC cl. I were also reported in LGMDR1 related to *CAPN3* mutations [9]. In children diagnosed with inflammatory myopathy mor than half of them showed mutations in *LMNA,* highlighting that diagnosis of both hereditary and acquired myopathy with inflammation may be challenging [38].

In respect to Duchenne muscular dystrophy (DMD), specific inflammatory features emerged as characteristic alterations on muscle biopsy tissues [7]. As such, DMD muscle fibers may contain epitopes targeted by autoreactive T cells with sarcolemmal MHC cl. I detected on myofibers [47]. Following this line of argumentation, there is an ongoing discussion regarding the diagnostic significance of MHC cl. I detection. Currently, a systematic study of muscular dystrophies with focus on MHC and complement remains an unmet need. Notwithstanding, the observed inflammatory features provide an intriguing link between genetic muscle disorders and associated immunopathologies.

### Sporadic late onset myopathy

Muscle biopsies from sporadic late onset nemaline myopathy (SLONM) patients are characterized by the presence of nemaline rods in the sarcoplasm of myofibers. Here, sarcolemmal MHC cl. I is observed in ~ 66% of cases, while only a marginal percentage of patients demonstrate MHC cl. II [86]. Complement deposits were found on both capillaries and the sarcolemma at varying quantities, irrespective of an association with monoclonal gammopathies [86].

### Autophagic vacuolar myopathies

Autophagic vacuolar myopathies (AVM) are a heterogeneous group of muscle disorders that share autophagic vacuoles as pathomorphological hallmark [53]. They share defective autophagy as common feature and include hereditary diseases, such as Pompe disease, Danon disease or X-linked myopathy with excessive autophagy and very rare disorders such as valosin-containing protein (VCP)-associated myopathy or matrin-3-related distal myopathy. Concurrently, acquired myopathies such as chloroquine/hydroxychloroquine toxicity may also present with autophagic vascular features [23, 44, 50]. The interplay between disrupted autophagy mechanisms and the consequent buildup of cellular debris constitutes a central aspect of the pathophysiology of these disorders. Despite their rarity, AVMs constitute a diagnostic challenge as they may exhibit deposition of C5b-9 along the sarcolemma and within vacuoles, as well as sarcolemmal MHC cl. I staining on myofibers [18, 23]. Currently, there is a knowledge gap regarding the distribution of MHC class II in AVMs. Although the identification of sarcolemmal C5b-9 and MHC cl. I suggest an inflammatory muscle pathology, the diagnosis should be approached with caution if cytoplasmic autophagic vacuoles are also present, particularly when coupled with clinical manifestations like cardiomyopathy and a gradually progressive disease trajectories.

## Search strategy and study identification

**Fig. 5:**
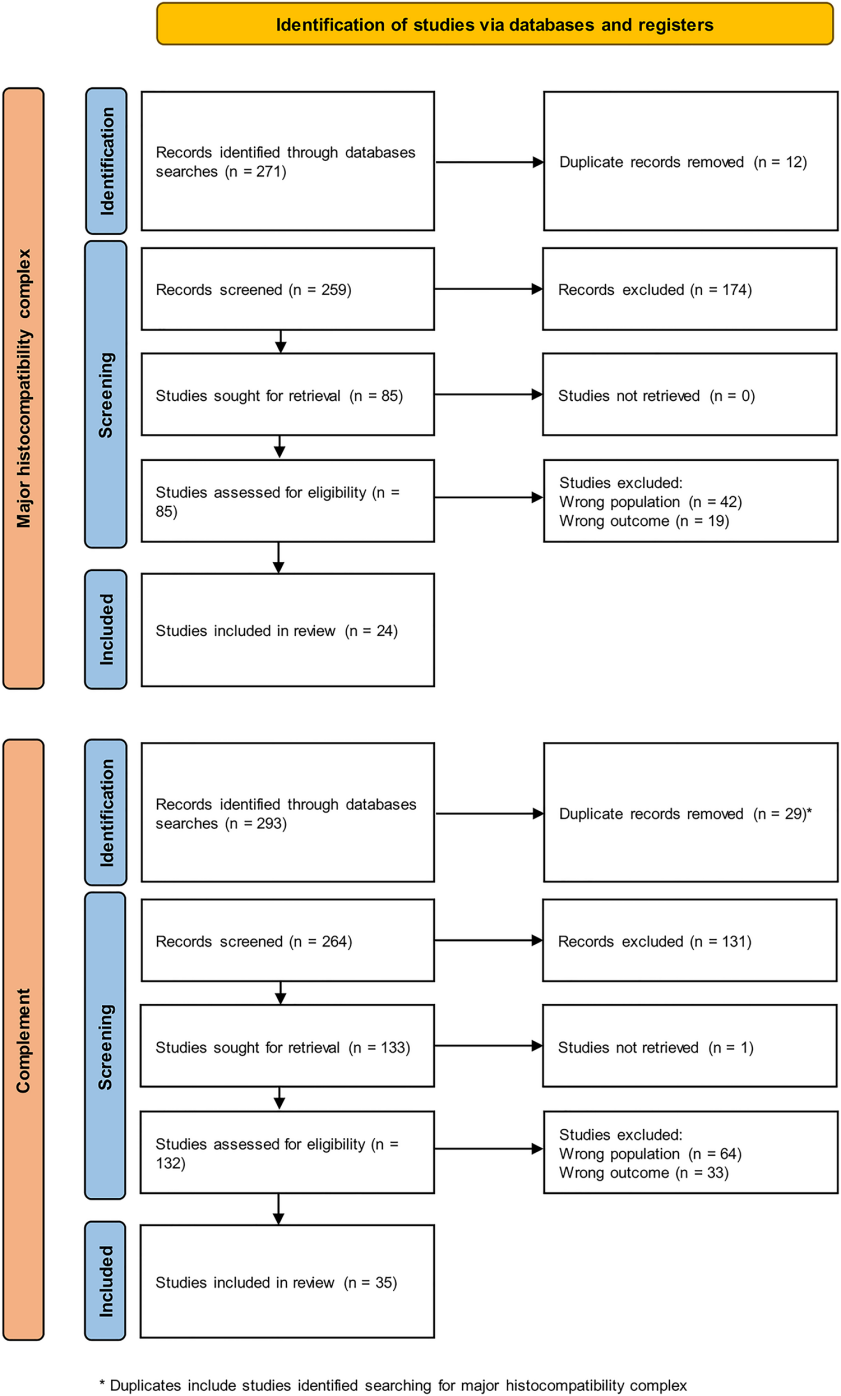
Preferred reporting items for systematic reviews and meta-analyses (PRISMA) flowchart describing the study search and inclusion process for this review. We conducted two separate searches for major histocompatibility complex and for complement patterns. Duplicate studies across these two searches were only included in the upper flowchart

For the algorithm proposed in this manuscript, we aimed to systematically assess the available literature. Our aim was to mitigate bias and consolidate the available research to underpin a strategy employing MHC and complement staining patterns. The study's inclusion and exclusion criteria adhered to the guidelines outlined in the Preferred Reporting Items for Systematic Reviews and Meta-Analyses (PRISMA) [61], as depicted in Fig. 5.

### Eligibility criteria

We considered all types of primary research including peer-reviewed publications, reports and posters. Preprints, commentaries, editorials, letters and book chapters were excluded. Only papers in English language were considered.

### Context

Study inclusion required that the population consists of at least of one subtype of IIM and diseased or non-diseased controls. As outcome measure, we assessed histopathological descriptions of major histocompatibility complex (MHC) or terminal complement (C5b-9).

### Search strategy

We identified relevant studies by searching the medical databases MEDLINE (PubMed), Web of Science and Google Scholar. The following strings were used as query: (1) (myositis OR idiopathic inflammatory myopathy) AND (major histocompatibility complex OR MHC) NOT review NOT animal study; (2) (myositis OR idiopathic inflammatory myopathy) AND complement NOT review NOT animal study. Our search was conducted on 10 October 2023. We used no publication date restrictions.

### Study extraction

Records identified after applying our search strategy were uploaded into reference manager Zotero (v6.0.27) and duplicates were removed. Titles and abstracts were screened by CN and CBS. Full text articles were obtained for abstracts that needed to be included or that appeared unclear. Studies without controls were excluded.

With this approach, we identified a total of 29 studies that provided data on the histomorphological appearance of MHC cl. I and II as well as terminal complement in IIMs. A shared limitation to the majority of studies was a lack of controls as well as comparative statistical approaches to determine diagnostic specificity and sensitivity. It is crucial to acknowledge these limitations when interpreting the proposed algorithm. Furthermore, these constraints underscore the pressing need for systematic, prospective assessments in the search for effective diagnostic markers for IIMs.

## A diagnostic algorithm based on MHC and complement patterns for IIM

**Fig. 6:**
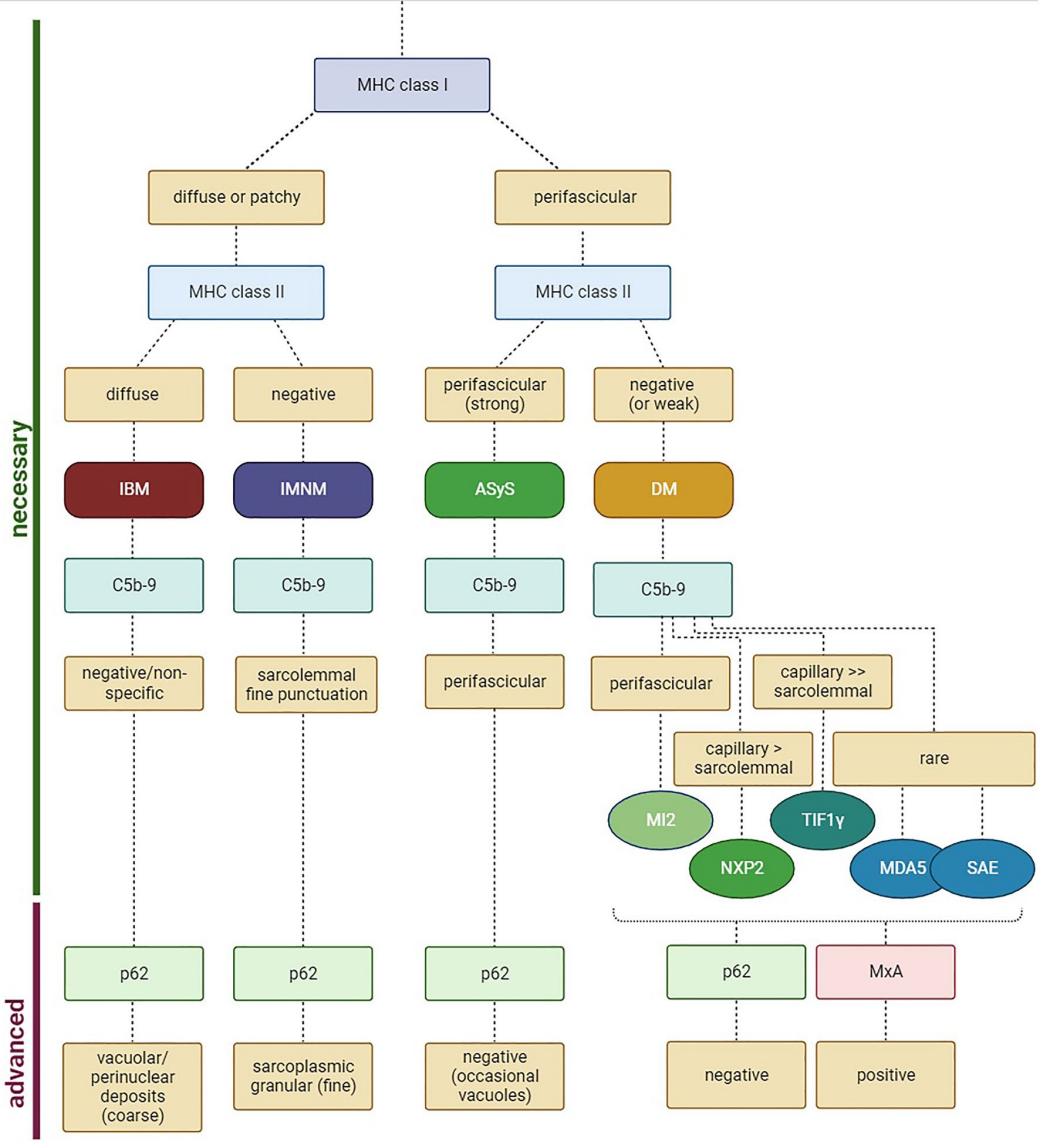
Proposed algorithm for distinguishing inflammatory idiopathic myopathies. To distinguish inclusion body myositis (IBM), immune-mediated necrotizing myopathy (IMNM), anti-synthetase syndrome (ASyS) and dermatomyositis (DM), we propose to stain for MHC class I, MHC class II and terminal complement (C5b-9). By observing these patterns, a panel of only three stainings may suggest a diagnosis. For further analysis, one may use p62 as marker of autophagy and MxA as a marker for a type I interferon response indicating DM as likely diagnosis. *MDA5* melanoma differentiation-associated protein 5; *MHC* major histocompatibility complex; *MxA* myxovirus resistance protein A; *NXP2* nuclear matrix protein 2; *SAE* small ubiquitin-like modifier-1 activating enzyme; *TIF1γ* transcriptional intermediary factor 1 gamma

MHC cl. I and -II and complement patterns differ across IIM entities. Here, we propose that MHC cl. I and -II in combination with complement may be employed to define muscle pathologies following a simplified algorithm (Fig. 6), allowing for a broad but sufficient subclassification that is required for current therapeutic approaches [16].

It is important to highlight that the proposed approach is founded on the limited scope of existing research. The rarity and heterogeneity of IIMs constitute a challenge for the conduction of prospective, large-scale investigations aimed at interrogating the specificity and sensitivity of diagnostic markers. The algorithm outlined in this manuscript is intended to provide a framework for future studies and to provide muscle pathologists with clues that can enhance diagnostic confidence, even when operating with limited data or resources. Further studies are imperative to provide the necessary evidence for a conclusive diagnostic algorithm and with this review we hope to stimulate such research.

Here, we propose to include MHC cl. I staining in the diagnostic work-up of IIMs as early step in order to distinguish established subtypes of this disease spectrum. A diffuse or patchy pattern of MHC cl. I suggests IBM or IMNM as underlying pathology, while a perifascicular pattern is observed in cases of ASyS or DM.

Subsequently, MHC cl. II staining enables further differentiation. MHC cl. II appears diffuse and strong in IBM, while IMNM is usually negative for MHC cl. II. Concurrently, ASyS features a strong perifascicular MHC cl. II pattern, while MHC cl. II typically appears negative or weakly positive, such as in cases of anti-Mi2-antibodies, in DM.

To improve diagnostic confidence, staining for C5b-9 may be employed. Here, IBM presents a negative or non-specific staining for C5b-9. IMNM, in contrast, features a sarcolemmal C5b-9 pattern with fine punctuation of a variable number of non-necrotic myofibers. ASyS demonstrates a perifascicular pattern of C5b-9 staining of myofibers. A similar pattern might be observed in anti-Mi2-antibody positive DM, constituting a diagnostic challenge. To resolve this challenge, additional staining for MxA is advised, given the high sensitivity and specificity of this marker in DM. Conversely, ASyS usually does not feature MxA positivity. Other serological groups of DM feature a capillary and to a lesser degree sarcolemmal pattern of C5b-9 in anti-NXP2-antibody and even more specifically anti-TIF1γ-antibody positive DM. In anti-MDA5-antibody and anti-SAE-antibody positive DM, C5b-9 staining on myofibers or capillaries is only rarely observed. Taken together, MHC cl. I, cl. II and complement (C5b-9) patterns may suggest an IIM subtype based on histopathological analysis. This assumption should be interpreted in the context of clinical and serological information.

For further analysis, p62 may be useful to highlight autophagy in muscle pathology. This staining may be particularly useful to differentiate between the fine granular and homogenous p62 pattern seen in IMNM linked to defective chaperone-mediated autophagy [29], and the pattern seen in IBM characterized by coarse, vacuolar staining of p62 in subsarcolemmal or perinuclear regions [12] (Supplemental Fig. 2a, b). In DM and ASyS, p62 is detected in a non-specific pattern.

## Complement in myasthenia gravis

Beyond the study of IIM, our current knowledge of complement also extends to other inflammatory neuromuscular disorders, such as myasthenia gravis (MG). Complement deposition was first described by Andrew Engel and the late Kiichi Arahata at the neuromuscular junction (NMJ) [27]. Since then, complement emerged as a pathophysiological hallmark in MG driving antibody-mediated destruction of the NMJ. The pathogenic role of complement is underlined by the clinical efficacy of complement inhibitors as demonstrated in recent phase 3 trials studying MG patients [36, 96]. Beyond serving as target for therapeutic strategies, complement might also serve as important diagnostic marker in MG. To understand how complement might serve diagnostic purposes, we shall first discuss the antibody patterns present in MG. Anti-acetylcholine receptor (AChR)-antibodies constitute the major serogroup of MG accounting for approximately 85% of cases [57, 58]. Anti-AChR-antibodies are primarily composed of immunoglobulin G1 (IgG1) and a minor proportion of IgG3 [78]. Anti-muscle specific kinase (MuSK)-antibodies account for approximately 5% of patients and are predominantly of the IgG4 subtype [72]. Less than 3% of MG patients harbor anti-low‐density lipoprotein receptor-related protein 4 (LRP4)-antibodies. Most anti-LRP4-antibodies belong to the IgG1 subclass [71]. The remaining (10–18%) of MG patients are considered (triple) seronegative (3SN). Although not without contestation, IgG4 antibodies are suggested to not induce complement activation, while IgG1 antibodies (and IgG2 and IgG3 to a lesser extent) trigger the classical complement cascade [19, 94]. Following this line of thought, it seems intuitive that antibodies belonging to the IgG1 subclass trigger complement activation and damage to the NMJ. Exemplified by anti-AChR-antibody MG, this concept has been validated on the histopathological level [35] and by the clinical efficacy of complement inhibitors [96]. In contrast, complement deposition can only be detected in a minority of anti-MuSK-antibody MG patients on a histopathological level [79]. Assessing the effect of complement inhibition on the course of anti-MuSK-antibody MG is difficult as conclusive data from clinical trials are currently lacking [51].

From a diagnostic viewpoint, the detection of pathogenic antibodies in conjunction with characteristic clinical presentation often enables accurate diagnosis in seropositive patients. However, seronegative MG patients can constitute a diagnostic and therapeutic challenge, particularly if the clinical phenotype is inconclusive or if patients are therapy-refractory to basic therapy, [17, 35]. To improve our understanding of seronegative MG, we recently studied external intercostal muscle biopsies from 13 3SN MG patients [35]. We identified CD56^+^ endplates and detected the presence of C5b-9 with variable intensity on all analyzed muscle specimens. Intriguingly, C5b-9 colocalized with IgG1 as well as C1q [35]. Together, these findings indicate that the currently unknown antibodies in 3SN MG might be of the IgG1 subclass mediating postsynaptic damage of the NMJ by inducing the classical complement pathway. Further studies are needed to provide the groundwork for therapeutic targeting of complement in seronegative MG. Still, as the presence of complement at the endplate is a highly specific finding for MG, histopathological analysis of (intercostal) muscle biopsies is a valuable approach to combat diagnostic uncertainty in seronegative MG. However, as muscle biopsies are invasive and therefore not a routine approach in MG, the additional diagnostic value must be assessed individually.

## Concluding remarks and future directions

Histopathological analysis remains the gold standard for diagnosis in many inflammatory neuromuscular disorders. To improve the availability of accurate histopathology, standardized and cost-effective workflows are required. These workflows should incorporate a straightforward algorithm that can be applied broadly.

The aim of this review was to highlight the diagnostic value of interpreting MHC and complement patterns in inflammatory muscle pathologies and relevant differential diagnoses. We suggest that MHC and complement can serve as a readily available set of IHC stains that can be used as biomarkers for disease classification. Minimizing the amount of required staining steps could be valuable in resource-limited settings and for design of diagnostic algorithms. It is important to highlight that clinical and laboratory information as well as conventional histology are needed to draw accurate conclusions from IHC. Concurrently, we also emphasize that while a biomarker might hold diagnostic significance, it does not necessarily imply direct pathophysiological importance—a point exemplified in the context of complement activation in IMNM. While complement deposition is a frequent feature of IMNM [2, 100], treatment with a C5 inhibitor did not appear effective [46], suggesting that complement activation may be a secondary feature to muscle fiber necrosis. This concept is reinforced by hereditary myopathies, such as LGMDR2 myopathy [15, 37], where complement activation accompanies muscle fiber necrosis and regeneration. This association hints at complement involvement potentially being a nonspecific feature following muscle injury. Thus, while complement markers can serve as diagnostic indicators, interpreting their pathophysiological significance requires careful consideration. To test the sensitivity and specificity of MHC and complement patterns in a diagnostic algorithm, prospective and adequately powered studies are required. We believe this approach to be useful to determine the necessary IHC steps required for accurate diagnosis when moving toward a standardized workflow for muscle pathologies.

### Supplementary Information

Below is the link to the electronic supplementary material.

Supplementary file1 (TIF 2641 KB)

Supplementary file2 (TIF 2639 KB)
