# Supplementary figures and images for: Complement and MHC patterns can provide the diagnostic framework for inflammatory neuromuscular diseases
Source: Acta Neuropathol. 2024 Jan 12;147(1):15. doi: 10.1007/s00401-023-02669-8 (PMC10786976; doi:10.1007/s00401-023-02669-8)

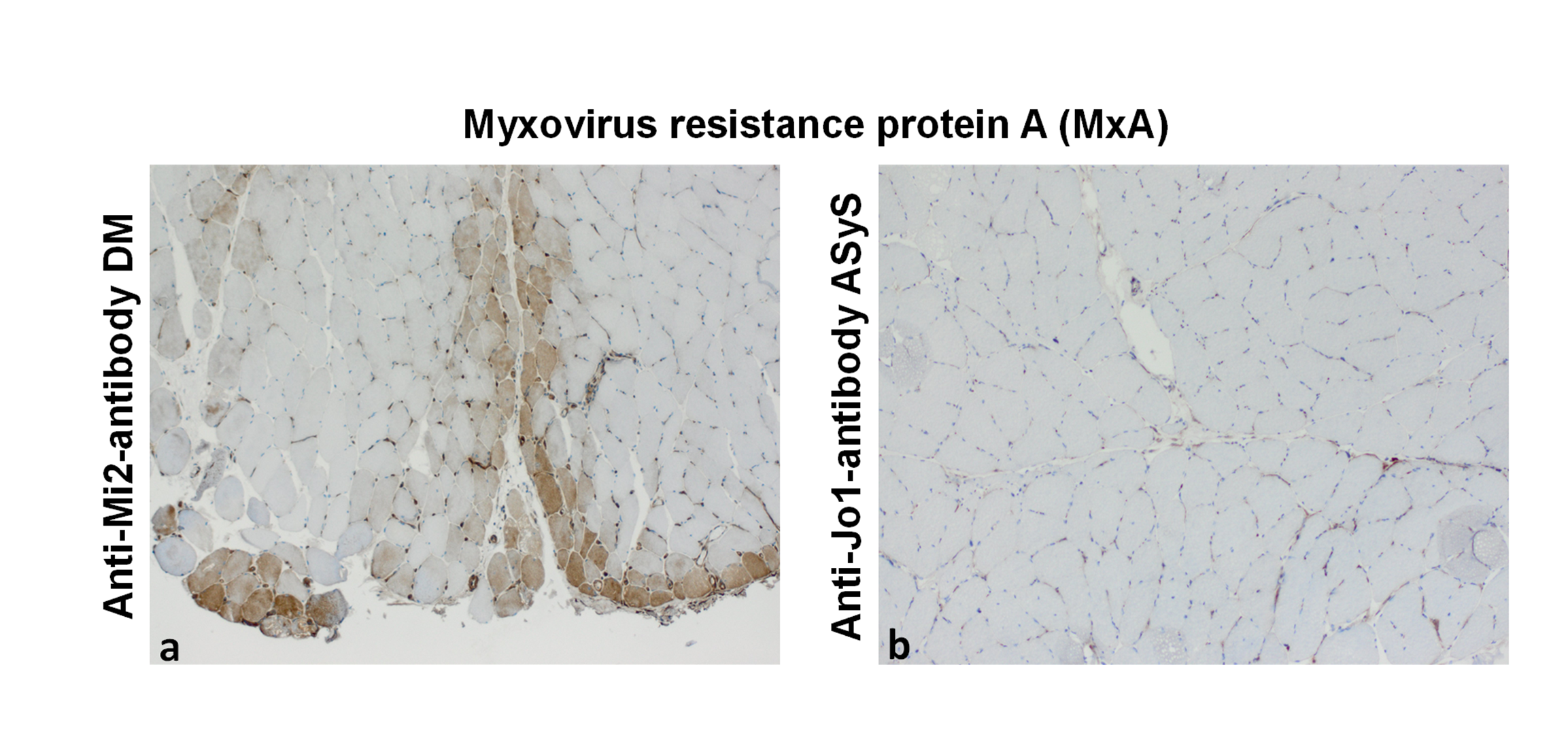

Supplement: Supplementary file 1 — Supplementary file1 (TIF 2641 KB) [file 401_2023_2669_MOESM1_ESM.tif]

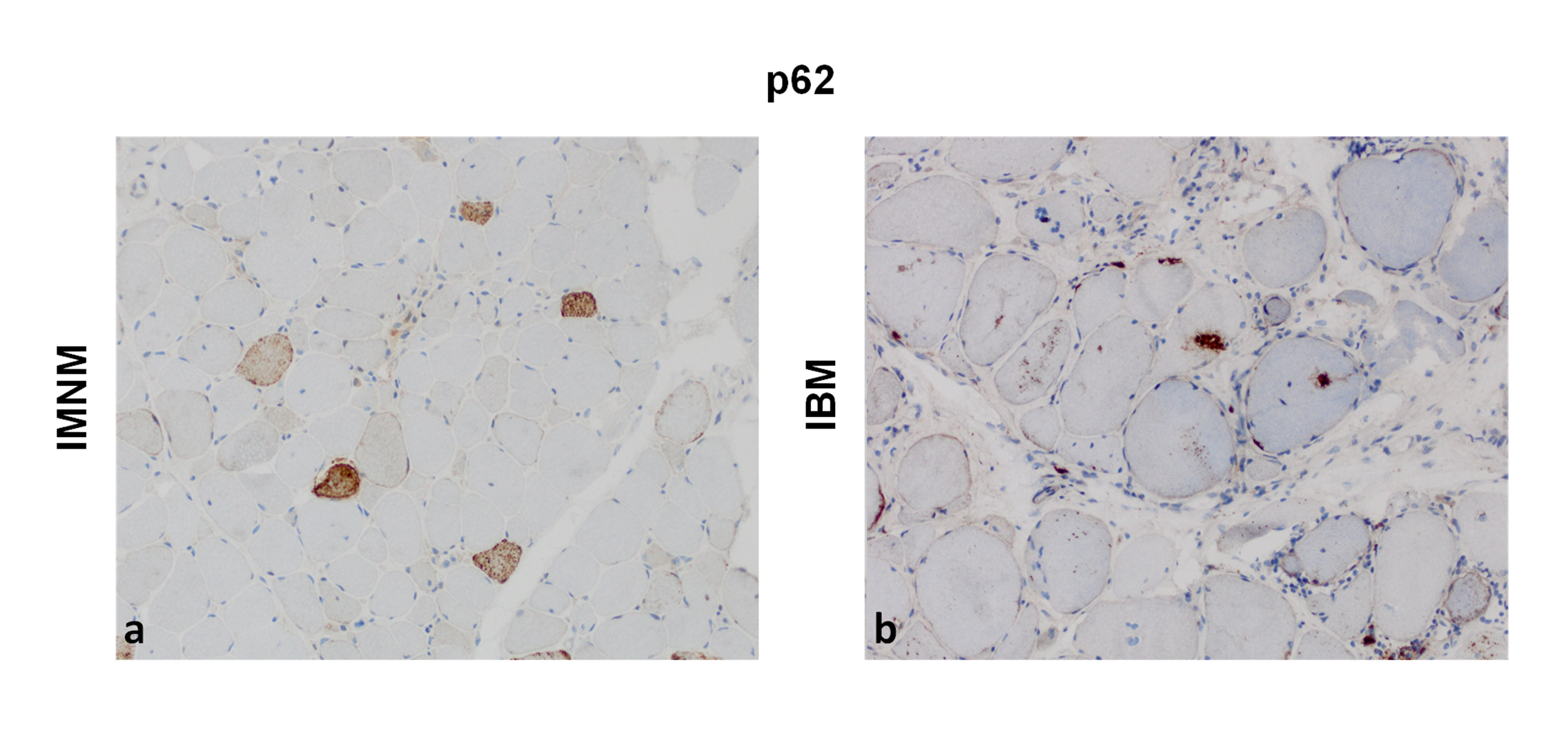

Supplement: Supplementary file 2 — Supplementary file2 (TIF 2639 KB) [file 401_2023_2669_MOESM2_ESM.tif]
